# Clinical and dermoscopic assessment of the efficacy of topical trichloroacetic acid 70% versus methoxsalen 0.2% paint in stable acral vitiligo

**DOI:** 10.1038/s41598-025-88811-w

**Published:** 2025-02-08

**Authors:** Ahmed R. Elshahed, Amr M. Ammar, Abdallah M. Ali, Mohamed L. Elsaie

**Affiliations:** 1 Department of Dermatology, Venereology and Andrology, Faculty of Medicine, Al- Azhar University, Cairo, Egypt; 2 Department of Dermatology, Medical Research and Clinical Studies Institute, National Research Centre, Giza, Egypt

**Keywords:** Vitiligo, TCA, Methoxsalen, Dermoscope, Health care, Medical research

## Abstract

Loss and absence of melanocytes due to a number of factors is responsible for vitiligo; known to be the commonest disorder of pigmentation. The aim of the study was to assess clinically and dermoscopically the efficacy of topical trichloroacetic acid 70% versus methoxsalen 0.2% paint in stable acral vitiligo. The patients were randomly divided into 2 groups. Group a (*n* = 35) received topical 0.2% methoxsalen every other day for 4 months duration with dermoscopic follow up while group b (*n* = 35) received received topical TCA 70% application at the clinic every two weeks for 4 months with dermoscopic follow up. The majority of subjects in both groups experienced either no or mild improvement. In TCA group, mean improvement was 4.0 ± 11.6% with range of 0–60% while in the methoxsalen group, mean improvement was 0.57 ± 3.3% with range of 0–20% (*p* = 0.051). The majority of patients reported poor satisfaction. Both modalities did not demonstrate a significant clinical nor dermoscopic response. TCA 70% had a lower effective rate than other studies probably due to resistance of acral vitiliginous lesions to treatment in comparison to other sites of the body. Further larger multi centre studies with different concentration and combination modalities are required to detect promising treatments for vitligo.

## Introduction

Vitiligo is an acquired pigmentary skin disorder by the absence of pigmentary cells from the epidermis that results in white macules and patches on the body. The condition is usually associated with few autoimmune disorders, with thyroid abnormalities are the commonest one^1^.

The etiology of vitiligo is unknown but there are different theories to explain its pathogenesis. Vitiligo presents clinically with signs and symptoms of white spots on the body distributed symmetrically and more obvious in people with dark skin^2^. A number of genetic, environmental and behavioral interactions are among the contributing factors for progression or remissions in vitiligo^3^.

The use of trichloroacetic acid (TCA) in the treatment of vitiligo is hasn’t been widely studied and only fewer studies exploited its efficacy as a therapeutic modality in vitiligo^4–8^. Despite not yet fully elucidated; induction of inflammation of viable melaoncytes followed by post inflammatory hyperpigmentation remains to be a proposed mechanism of TCA for activation of repigmentation in vitilgo patches^9^.

Methoxsalen is a psoralen produced naturally by various plants (e.g. celery, parsnips, limes, figs, and others) found in both temperate and tropical regions. The use of topical methoxsalen in the treatment of vitiligo involves applying the medication directly to the affected areas of the skin. Methoxsalen works by sensitizing the skin to ultraviolet A (UVA) light^10^.

The aim of the study was to assess clinically and dermoscopically the efficacy of topical TCA 70% versus methoxsalen 0.2% paint in stable acral vitiligo.

## Patients and methods

All patients (or guardians if needed) were informed about the nature and the possible risks of the study and the details of the procedure and asked to provide written informed. Written informed consent was obtained from every patient at the recruitment.

Patients from 10 to 50 years of age and both sexes were included if they complained of stable acral vitiligo and have not received any form of vitiligo treatment during the last 3 months before recruitment. Stable vitiligo was determined if at least two items were identified: VIDA score ≤ 0; clinical feature of lesions with clear edges or signs of repigmentation; no Koebner phenomenon within 1 year; white lesion with sharply clear borders, smaller than or equal to the visual area under Wood’s light.

Exclusion criteria included pregnant or lactating females, Patients who had a history of photosensitivity, diseases exacerbated by sunlight, keloids or hypertrophic scars, were excluded, and those who showed sensitivity to the products were excluded from the study. Patients with active vitiligo were excluded by VIDA score, VSAS Score and dermoscopic scoring for stability in vitiligo (BPLeFoSK criteria). Moreover Subjects with cognitive impairment, present psychiatric disorders were also excluded.

**Every patient was subjected to**:

(I) ** Questioning about**:

1. Personal history including; name, age, sex, and residence.
2. Present history including; onset, course, and duration of vitiligo.
3. Past history of any chronic illness or associated autoimmune disease.
4. Family history of vitiligo, premature graying of hair, or other general diseases.
5. Drug history as previous phototherapy, oral or topical medications.

(II) **General and Dermatological examination**:

- To exclude dermatological diseases other than vitiligo.
- All patients were subjected to carful dermatological examination in order to define the type and distribution of vitiligo, exclusion of any other skin problem as Koebners phenomenon.
- Wood’s lamp (Derma India, Chennai, India) and Dermoscopic (DermLite DL4, 3 Gen, USA) examination were performed to confirm the diagnosis.

(III) **Treatment protocol**.

The patients were randomly divided into 2 groups. Group a (*n* = 35) received topical 0.2% methoxsalen every other day for 4 months duration with dermoscopic follow up while group b (*n* = 35) received received topical TCA 70% application at the clinic every two weeks for 4 months with dermoscopic follow up.

In group 1 patients were instructed to apply methoxsalen every other day to the depigmented skin patch as a thin film sufficient to cover the affected area and then to rub it gently three to four times while in group 2; TCA was applied in clinic every two weeks. Skin was first cleaned with alcohol to remove the residual oils and scales until it felt dry then TCA solution was applied using two to four cotton-tipped applicators to the skin until frosting, or visible blanching, of the skin is achieved. 1 to 3 passes were usually sufficient to achieve frosting.

(IV) **Evaluation**.

All patient photographs were taken and documented by an expert before and upon completion of the treatment. Efficacy was blindly assessed by two independent expert dermatologists depending on the percentage of repigmentation. Repigmentation was evaluated by a quartile grading scale (classified as “0, absent” (0%), “1, poor” (1–25%), “2, moderate” (26–50%), “3, good” (51–75%), and “4, excellent” (> 75%). Dermoscopic repigmentation was classified into four patterns, including perifollicular, marginal, diffuse and mixed patterns, which were identified using all study images. Adverse events and complications were recorded in every visit.

(V) **Follow-up and adverse events**.

Standard images were taken by a 16-megapixel digital camera (Canon Power Shot A3400 IS 16 MP digital camera; Tokyo; Japan) constantly at baseline, after 4weeks, 8weeks and after three months of treatment. Follow up including: Comparing the photographs before and after therapy; evolution of clinical response included degree of pigmentation and possible adverse effects including erythema, burning sensations and blister formation.

### Statistical analysis of the data

Data were fed to the computer and analyzed using IBM SPSS software package version 20.0. (Armonk, NY: IBM Corp) Qualitative data were described using number and percent. The Shapiro-Wilk test was used to verify the normality of distribution Quantitative data were described using range (minimum and maximum), mean, standard deviation, median and interquartile range (IQR). Significance of the obtained results was judged at the 5% level.

## Results

The baseline characteristics of the subjects in both groups were significantly matched. The median age for the TCA group was 19 years (IQR 15–25 years) while for the methoxsalen group was 25 years (IQR 17–37 years). Group 1 had 15 (42.9%) males and 20 (57.1%) females group 2 was comprised of 13 (37.1%) males and 22 (62.9%) females. The median age of onset of disease among the TCA group was 14 years with IQR of 11–30 years while in the methoxsalen group was 20 years with IQR of 12–30 years. In TCA group, median disease duration was 4 years with IQR of 2–6 years while in the methoxsalen group, median disease duration was 5 years with IQR of 1–7 years. The TCA group included 12 (34.3%) type III and 23 (65.7%) type IV Fitzpatrick skin phototype while the methoxsalen group included 16 (45.7%) III and 19 (54.3%) IV Fitzpatrick skin phototype (*p* = 0.768). In TCA group, there were 16 patients (45.7%) with hand lesion and 19 patients (54.3%) with foot lesion compared to 17 patients (48.6%) with hand lesion and 18 patients (51.4%) with foot lesions among the methoxsalen group (Tables 1 and 2).

**Table 1:** Comparison of demographic data between studied groups.

		TCA (*N* = 35)		Methoxsalen (*N* = 35)		Stat. test	*P*-value
Gender	Male	13	37.1%	15	42.9%	X^2^ = 0.23	0.626 NS
	Female	22	62.9%	20	57.1%		
Age (years)	Median	19		25		MW = 554.5	0.495 NS
	IQR	15–35		17–37			
Age of onset (years)	Median	14		20		MW = 564	0.568 NS
	IQR	11–30		12–30			
Disease duration (years)	Median	4		5		MW = 566.5	0.586 NS
	IQR	2–6		1–7			
Family history	Negative	21	60%	18	51.4%	X^2^ = 0.52	0.470 NS
	Positive	14	40%	17	48.6%		

MW, Mann Whitney U tests. X^2^, Chi-square test. NS, p-value > 0.05 is considered non-significant.

**Table 2:** Comparison of clinical data between studied groups.

		TCA (*N* = 35)		Methoxesalin (*N* = 35)		Stat. test	*P*-value
Lesion side	Right	17	48.6%	35	100%	**X**^**2**^ **= 24.2**	**< 0.001 HS**
	Left	18	51.4%	0	0%		
Lesion site	Hand	16	45.7%	17	48.6%	X^2^ = 0.057	0.811 NS
	Foot	19	54.3%	18	51.4%		
Skin type	Type III	12	34.3%	16	45.7%	X^2^ = 0.95	0.329 NS
	Type IV	23	65.7%	19	54.3%		

Significant values are in [bold].

HS, p-value < 0.001 is considered highly significant. X^2^, Chi-square test.

NS, p-value > 0.05 is considered non-significant.

Clinical improvement based on the quartile grading scale at the end of treatment did not show any statistically significant difference between groups. The majority of subjects in both groups experienced either no or mild improvement. In TCA group, mean improvement was 4.0 ± 11.6% with range of 0–60% while in the methoxsalen group, mean improvement was 0.57 ± 3.3% with range of 0–20% (*p* = 0.051). The majority of patients in the TCA group reported poor satisfaction with 29 not satisfied patients (82.9%), 4 partially satisfied patients (11.4%) while in the methoxsalen group; 34 (97.1%) of the included subjects reported no satisfaction (Table 3).

**Table 3:** Comparison of clinical outcome between studied groups.

		TCA (*N* = 35)		Methoxsalen (*N* = 35)		Stat. test	*P*-value
Improvement (%)	Mean ± SD	4.0 ± 11.6		0.57 ± 3.3		MW = 526	0.051 NS
	Range	0–60		0–20			
Patient’s satisfaction	Not satisfied	29	82.9%	34	97.1%	X^2^ = 4.19	0.123 NS
	Partially satisfied	4	11.4%	1	2.9%		
	Satisfied	2	5.7%	0	0%		
Pattern of repigmentation	No repigmentation	29	82.9%	34	97.1%	X^2^ = 0.95	0.329 NS
	Marginal pigmentation and appearance of reticular network	3	8.6%	1	2.9%		
	Perifollicular pigmentation and appearance of reticular network	1	2.9%	0	0%		
	Mixed	2	5.7%	0	0%		

X^2^, Chi-square test. NS: p-value > 0.05 is considered non-significant.

**Fig. 1:**
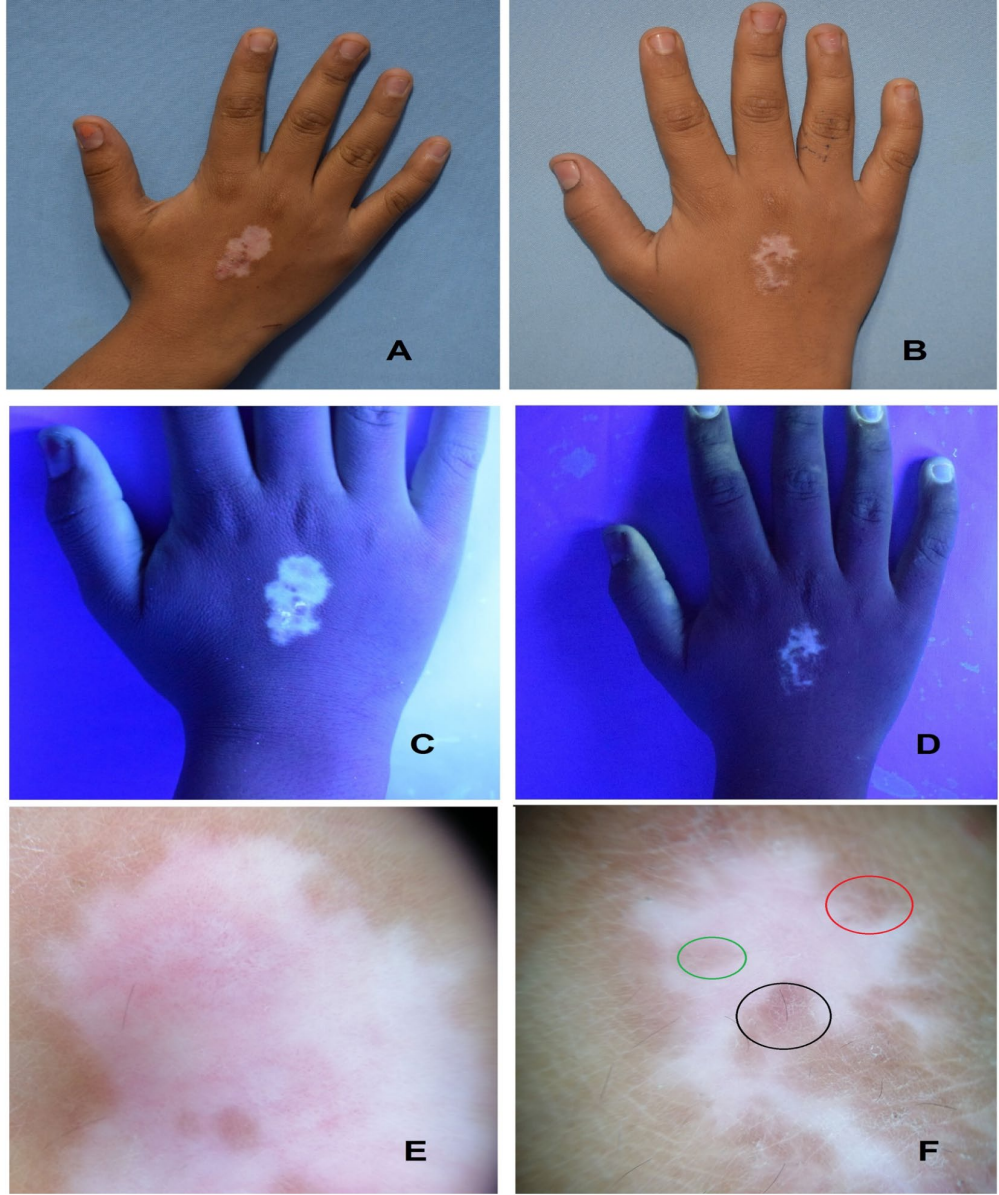
Clinical (A, B); Woods light (C, D) and dermoscopic (E, F) images showing appearance of the reticular network (black circle), marginal pigmentation, perifollicular pigmentation (mixed re-pigmentation patterns; red and green circles) before and after 3 months treatment with TCA 70%.

**Fig. 2:**
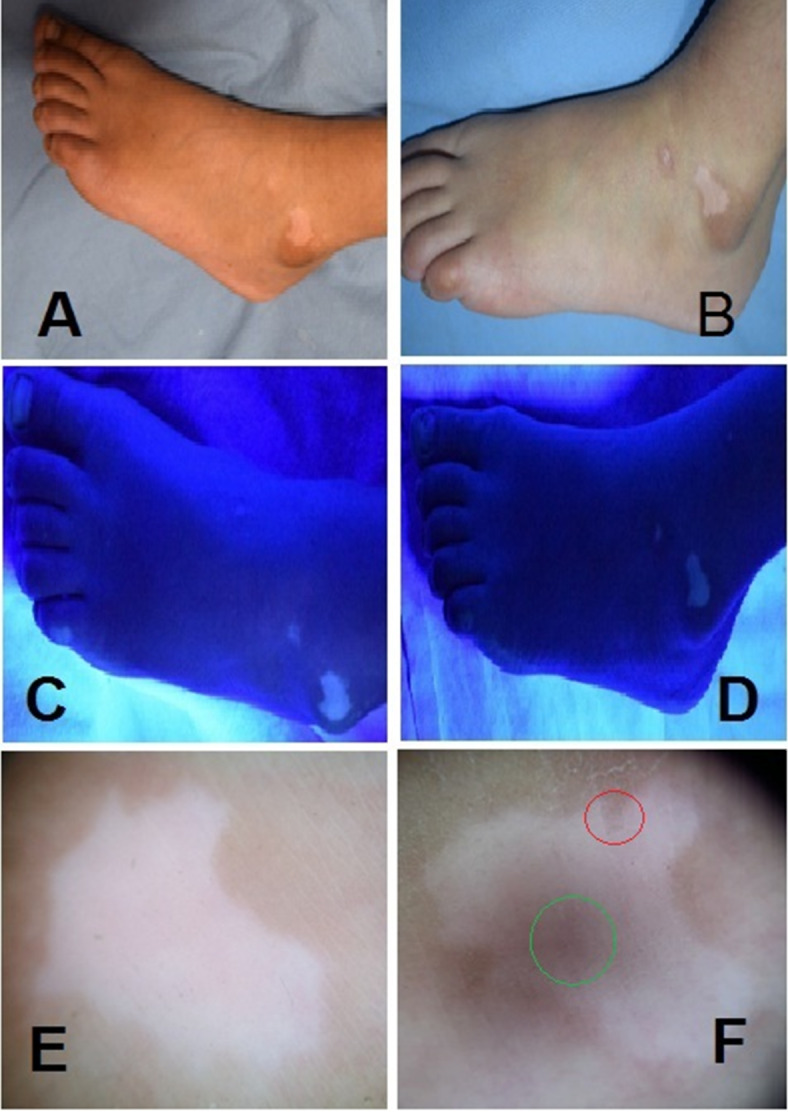
Clinical (A, B); Woods light (C, D) and dermoscopic (E, F) images showing appearance of the reticular network (green circle) and marginal pigmentation (red circle) before and after 3 months treatment with methoxsalen 0.2% paint.

As regard re-pigmentation pattern for the TCA group, there were 29 patients (92.9%) with no repigmentation, 3 patients (8.6%) with marginal pigmentation and appearance of reticular network, 1 patient (2.9%) with perifollicular pigmentation and appearance of reticular network and 2 (5.7%) patients only demonstrated mixed patterns. In the methoxsaen group, there were 34 patients (97.1%) with no repigmentation and 1 patient (2.9%) with marginal pigmentation and appearance of reticular network. The patterns of repigmentation (marginal, diffuse and perifollicular) did not significantly differ among subjects of both groups (*p* = 0.329) (Figs. 1, 2; Tables 3 and 4).

**Table 4:** Relation between clinical improvement and pattern of pigmentation dermoscopically in both groups.

		Clinical improvement				Total	*P* value
		N0	Mild	Moderate	Good		
Pattern of repigmentation dermoscopically	No re-pigmentation	63	0	0	0	63	< 0.001*
		90.0%	0.0%	0.0%	0.0%	90.0%	
	Marginal pigmentation and appearance of reticular network	0	4	0	0	4	
		0.0%	5.7%	0.0%	0.0%	5.7%	
	Perifollicular pigmentation and appearance of reticular network	0	1	0	0	1	
		0.0%	1.45%	0.0%	0.0%	1.45%	
	Mixed pattern of repigmentation	0	0	1	1	2	
		0.0%	0.0%	1.45%	1.45%	2.8%	
Total		63	5	1	1	70	
		90.0%	7.1%	1.45%	1.45%	100.0%	
X^2^ = 100							
Measure of association (Eta) = 0.98							

Significant at p value 0.05 ×^2^ mean chi square.

**Fig. 3:**
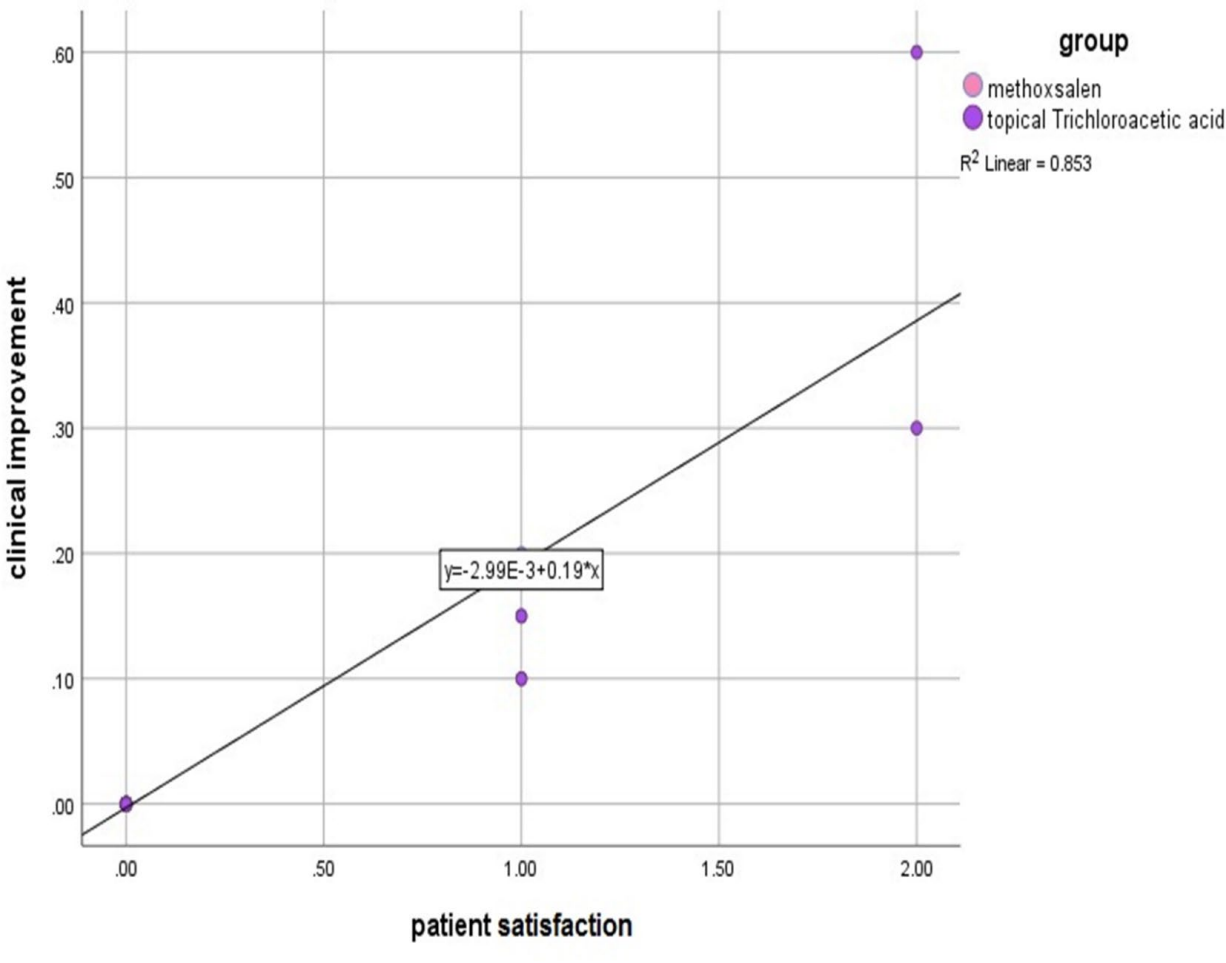
Scatter plot show correlation between patient satisfaction and clinical improvement.

Mild side effects were reported and treatments were significantly tolerated in both treatment groups. A number of cases complained of irritation or desquamation while only 2 cases complained of vesicles in the TCA groups that faded with no other serious events recorded. A positive correlation between clinical improvement and patient satisfaction was determined (*r* = 0.92; *p* < 0.01) while no significant correlation was found with duration of disease, age of onset and skin type (Fig. 3).

## Discussion

The management of vitiligo becomes challenging considering its complex etiopathogenesis. There is no definite cure available for vitiligo requiring more investigations with different modalities of treatment for optimal results. Acral vitiligo of the hands and feet is considered by many authors as the most challenging site for treatment and is usually resistant to many therapeutic modalities, including the most aggressive ones. This may be attributed to the lower hair follicle and melanocyte density.

In the current study, clinical improvement based on the quartile grading scale at the end of treatment did not show any statistically significant difference between groups. The majority of subjects in both groups experienced either no or mild improvement.

One study evaluating the efficacy and safety of TCA, in different concentrations, for the treatment of stable localized vitiligo. The study included 100 patients with acral/nonacral stable vitiligo. Trichloroacetic acid was applied, as a monotherapy, to the vitiliginous patches at different concentrations according to the treated site every 2 weeks until complete repigmentation or for a maximum of 6 treatment sessions. Follow-up was done every month for 6 months to detect any recurrence. Eyelid vitiligo showed the highest response to TCA treatment (excellent response in 80% of cases), followed by the face, trunk, and extremities. Lower response rates were noticed in the hands and feet vitiligo. Adverse effects were transient and insignificant in few patients^4^.

A comparative survey by Khater et al.^5^ enrolled 32 patients with vitiligo; group 1 was treated by microneedling, followed by TCA 70%, and group 2 was treated by intradermal 5-FU injection. They demonstrated the improvement was good to excellent (repigmentation > 50%) in 43.8% of patients in each group.

Puri and Puri^6^ evaluated the use of 100% TCA in 15 patients with stable nonacral vitiligo and reported marked repigmentation in 66.6% of the studied patients. Discrepancies could be attributed to the different concentration of TCA used besides they used psoralen and ultraviolet A after wound healing from TCA application.

Hunter and colleagues compared TCA 15% and 25% in the treatment of vitiliginous patches in 10 patients with nonsegmental, nonacral vitiligo (4 sessions, 1 week interval) followed by 48 sessions of NBUVB as a combination therapy. They reported moderate-to-marked repigmentation (60–100%) in 80% of the patients who received TCA 15%, and 60% of the patients who received TCA 25%. Discrepancies in results could be attributed to the different concentrations of TCA used besides combination treatment used in contrary to the monotherapy used in the current study^7^.

One study compared dermapen versus TCA 50% for treating idiopathic guttate hypomelanosis (IGH) macules and demonstrated that 65 out of 72 TCA 50% treated IGH macules showed significant repigmentation^11^. Moreover; TCA (100%) was shown to reconstruct a vitiligo lesion involving the lip in an adult female vitiligo patient^12^.

El Mofty and colleagues compared the use of TCA (15% and 25%) versus ablative fractional co2 laser versus microneedling with dermapen in the treatment of 30 patients with stable nonacral vitiligo (10 patients per group) and demonstrated higher response rates among the TCA peel group^8^.

Another study compared combination of microneedling and TCA (30% on the face and 50% on other sites) versus microneedling and 5 FU versus microneedling and pimecrolimus in 75 stable vitligo patients (25 assigned to each group). In all the groups, the procedure was done every 2 weeks until complete repigmentation was achieved or for a maximum of six treatment sessions. The difference between the three groups was statistically significant in favor of the combined microneedling and TCA^13^.

Acral vitiliginous lesions, located on the wrist, hand, ankle, and foot, are often resistant to medical therapies, probably due to their lower content of melanocytes and hair follicles, which harbor melanocyte stem cells. Moreover, their response to surgical treatment is often unsatisfactory, probably due to the continuous mobility of these sites. Due to the tissue structure and repeated friction experience, lesions of acral vitiligo have relatively thicker stratum corneum, which likely affects the penetration of topical drugs as well^14^.

Our study had some limitations; the relatively small sample size. In addition, selection bias, as the patients were primarily recruited from patients who attended to our university clinic only. Utilizing a multicenter approach would improve generalizability of the results, a larger sample size, and, consequently, improved efficiency Another limitation was the short follow up as well as using treatment for one type of vitiligo (Acral type).

Trichloroacetic acid (TCA) demonstrated a better response to methoxsaen though non effective or satisfactory among both modalities in the current study. TCA 70% had a lower effective rate than other studies probably due to resistance of acral vitiliginous lesions to treatment in comparison to other sites of the body. Moreover, the discrepancies observed in the family history findings as well as genetic variances as well as different concentrations of TCA and combination treatments used across these various studies could have attributed to the divergence in results. To achieve ideal therapeutic efficacy, a future treatment rational using larger multicenter studies and varied concentrations and enhanced delivery approaches is essential to explore the promising potential and improved outcomes of the above used treatments.

## Data Availability

The data that support the findings of this study are available from the corresponding author upon reasonable request.

## Abbreviations

TCA: Trichloroacetic acid
VIDA: Vitiligo disease activity
IQR: Inter quartile range

## Code availability

All authors confirm all data and materials as well as softwareapplications or custom code support their published claims andcomply with field material.
